# *Helicobacter pylori* CagA induces tumor suppressor gene hypermethylation by upregulating DNMT1 via AKT-NFκB pathway in gastric cancer development

**DOI:** 10.18632/oncotarget.7125

**Published:** 2016-02-02

**Authors:** Bao-gui Zhang, Lei Hu, Ming-de Zang, He-xiao Wang, Wei Zhao, Jian-fang Li, Li-ping Su, Zhifeng Shao, Xiaodong Zhao, Zheng-gang Zhu, Min Yan, Bingya Liu

**Affiliations:** ^1^ Shanghai Key Laboratory of Gastric Neoplasms, Department of Surgery, Shanghai Institute of Digestive Surgery, Ruijin Hospital, Shanghai Jiao Tong University School of Medicine, Shanghai, People's Republic of China; ^2^ Affiliated Hospital of Jining Medical University, Jining, People's Republic of China; ^3^ Department of Microbiology, Shanghai Jiao Tong University School of Medicine, Shanghai, People's Republic of China; ^4^ Bio-ID Center, School of Biomedical Engineering, Shanghai Jiao Tong University, Shanghai, People's Republic of China

**Keywords:** gastric cancer development, H. pylori CagA, DNMT1, hypermethylation, AKT-NF-kB pathway

## Abstract

Methylation of CpG islands in tumor suppressor gene prompter is one of the most characteristic abnormalities in *Helicobacter pylori* (HP)-associated gastric carcinoma (GC). Here, we investigated the pathogenic and molecular mechanisms underlying hypermethylation of tumor suppressor genes in HP induced GC development. We found that tumor suppressor genes hypermethylation, represented by MGMT, positively correlated with CagA in clinical specimens, gastric tissues from HP infected C57 mice and GC cell lines transfected by CagA or treated by HP infection. CagA enhanced PDK1 and AKT interaction and increased AKT phosphorylation. The P-AKT subsequent activated NFκB, which then bound to DNMT1 promoter and increased its expression. Finally, the upregulated DNMT1 promoted tumor suppressor genes hypermethylation with MGMT as a representative. In conclusion, CagA increased tumor suppressor genes hypermethylation via stimulating DNMT1 expression through the AKT-NFκB pathway.

## INTRODUCTION

A causal relationship between HP and GC was first postulated by Marshall and Warren in 1983 [1]. Recent studies indicate that both intestinal and diffuse types of GC are strongly associated with HP infection [2]. Most (89%) HP contain a contiguous cag pathologinicity island (cag-PAI), which encodes a known virulence factor CagA [3]. It has been reported that CagA plays an important role in GC carcinogenesis as an oncoprotein [3]. Besides, the internal organization of the cag-PAI appears to be highly conserved [4, 5]. It is known that HP has contact-dependent mechanisms to interact with and modify epithelial cells, including a type IV secretion system (TFSS) that injects CagA into host cells [6]. CagA has multiple effects on epithelial cells, including apical junction modification and cell polarity perturbation [7]. Although the exact mechanism of HP-associated GC is still elusive, long-standing bacterial infection and prolonged chronic inflammation are thought to generate a carcinogenic environment that gradually causes epigenetic reprogramming of host cells in the stomach [2].

Alteration of epigenetic information is involved in the development of many cancers and other diseases [8, 9]. DNA methylation is contributing mechanism of epigenetic transmission. Aberrant hypermethylation of promoter region CpG islands of tumor suppressor gene is involved to a greater extent in carcinogenesis in the stomach than in other human tissues or organs [10]. Kang and his group investigated the methylation profile in multistep lesions of the stomach and determined the methylation frequency of 12 genes, including O6-methylguanine-genes DNA methyltransferase (MGMT). They demonstrated that hypermethylation of certain tumor suppressor occurred in GC carcinogenesis and accumulated during progression of the gastric lesion along the multistep carcinogenesis pathway [11]. The gene MGMT has a CpG island within its promoter and thus its expression is significantly regulated by DNA methylation [12]. Chan *et al.* first demonstrated HP-associated hypermethylation in gastric epithelia, which was further verified in a recent study [13, 14]. Ushijima *et al.* compared methylation levels of eight regions in HP positive and HP negative individuals, and found that methylation levels of several CpG islands in HP-positive individuals were 5.4 to 303-fold higher than the corresponding levels in the HP-negative GC individuals [15]. Interestingly, previous studies have noted that methylated *MGMT* was significantly associated with CagA^+^ HP infection (*p* < 0.035) [14]. Development of DNA methylation biomarkers (MGMT) for the purposes of cancer screening, cancer risk assessment, and chemotherapy sensitivity prediction is currently evaluated in clinical trials [14]. However, despite this clear correlation between DNA methylation and HP infection, the mechanism by which HP causes hypermethylation of tumor superessor genes is unknown.

DNA methylation was mainly regulated by DNA methyltansferase family (DNMTs) [10], so we presumed that HP regulated suppressor gene promoter hypermethylation through DNMTs. A potential candidate methyltansferase is DNMT1, which has been widely implicated in the malignant transformation of various cancers [16]. In particular, DNMT1 is frequently overexpressed in cancers and contributes significantly to cancer-associated epigenetic silencing of tumor suppressor genes [17].

In this study, we found that CagA down regulated the MGMT expression by inducing hypermethylation in its promoter region, suggesting that CagA might induce gastric carcinogenesis by causing hypermethylation of tumor suppressor genes, with the MGMT as a representative.

## RESULTS

### *MGMT* was hypermethylated and its expression was downregulated in CagA+ tissues

**Figure 1:**
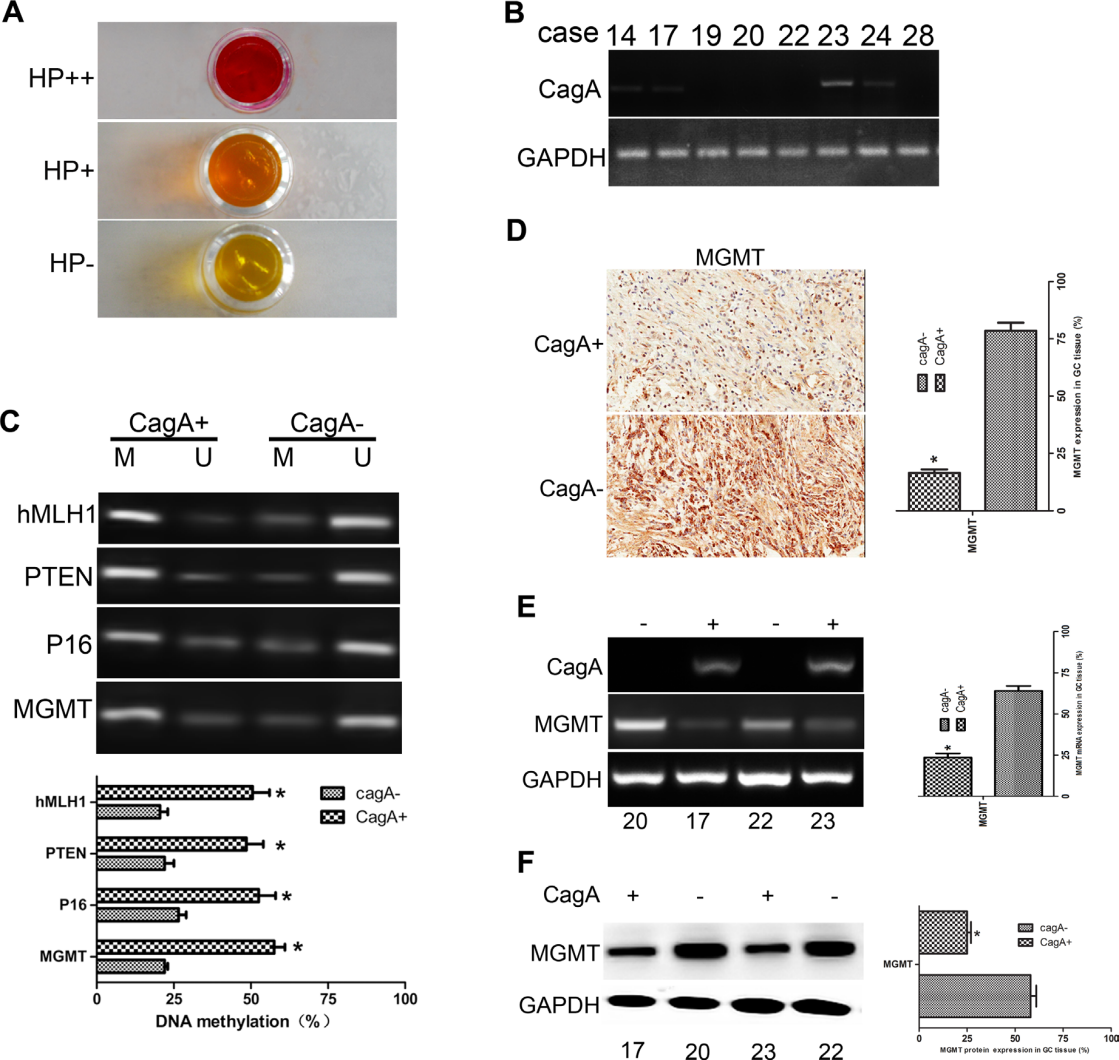
*MGMT* gene methylation and its expression in HP-associated GC tissues (**A**) Rapid urease test (RUT) is used to group clinical tissues into two categories: HP+ (pink and purple) and HP− (yellow). (**B**) RT-PCR of *CagA*. HP+ tissues are further subjected to RT-PCR to amplify CagA. HP+ tissues are classified as CagA+ or CagA−. (**C**) MSP of some conventional cancer suppressor genes including *MGMT* in HP+ tissues. Gene methylation increases sharply in CagA+ tissues compared with CagA− tissues (*p* < 0.05). (**D**) Immunohistochemical staining of MGMT in HP+ tissues. A notable loss of MGMT is found in CagA+ compared with CagA− tissues. (**E** and **F**) mRNA and protein levels of MGMT. RT-PCR and WB are applied to further evaluate MGMT expression. Consistent with results shown in (D), there is a significant difference in MGMT expression between CagA+ and CagA− tissues. The graph represents densitometric analysis of the bands obtained for each signal. Results are expressed as the relative expression compared with control cells (*p < 0.05). Each value is the mean ± SD of three experiments.

To investigate epigenetic mechanisms of HP induced GC, promoter methylation of tumor suppressor genes, represented by MGMT, was detected by MSP in GC tissues. We grouped surgically resected GC tissues into HP+ and HP− according to the standard rapid urease test (RUT) (Figure 1A). HP+ tissues were then subjected to RT-PCR to amplify CagA, and those tissues in which CagA could be amplified were classified as CagA^+^ (Figure 1B). Methylation specific PCR (MSP) analysis of GC related tumor suppressor genes was performed on DNA samples extracted from frozen tissues (Figure 1C). MGMT was selected to be the representative gene. We found that the average methylation degree of the *MGMT* gene in CagA^+^ samples was significantly higher than that in CagA^−^ samples (*p* < 0.05) (Figure 1C). Similarly, the ratio of GC tissues with undetectable levels of MGMT was significantly higher in CagA^+^ samples than that in CagA^−^ samples (15/22 vs 4/11, *p* < 0.05; Figure 1D and Table 1). With regard to the relationship between *MGMT* methylation and MGMT expression, a lower expression level was only observed in those samples showing a high degree of methylation (*P* < 0.05) (Figure 1D–1F).

**Table 1:** Relationship between MGMT expression, MGMT gene methylation, DNMT1 expression and clinicopathologic parameters in GC

Clinicopathologic Parameters (*n* = 33)	*MGMT-MPS* (++/±)*n*	*P*	*MGMT-IHC* (++/±)*n*	*P*	*DNMT1-IHC* (++/±)*n*	*P*
HP (CagA)						
Positive	17/5	0.009	7/15	0.031	18/4	0.049
Negative	3/8		8/3		5/6	
Age (years)						
≤ 59	10/5	N	7/8	N	12/3	N
> 59	10/8		8/10		11/7	
Gender						
Male	12/8	N	9/11	N	13/7	N
Female	8/5		6/7		10/3	
Bormann Type						
I–II	4/3	N	4/3	N	6/1	N
III–IV	16/10		11/15		17/9	
Location						
Middle/Proximal	7/5	N	5/7	N	8/4	N
Distal	13/8		10/11		15/6	
Diameter (cm)						
≤ 5	11/8	N	9/10	N	13/6	N
> 5	9/5		6/8		10/4	
Histologic type						
Intestinal	8/5	N	5/8	N	9/4	N
Diffuse	12/8		10/10		14/6	
Depth of invasion						
T1, T2	6/4	N	4/6	N	8/2	N
T3, T4	14/9		11/12		15/8	
Lymph-node metastasis						
No	7/4	N	5/6	N	8/3	N
Yes	13/9		10/12		15/7	
Differentiation						
High/Middle	8/4	N	5/7	N	8/4	N
Moderate/Low	12/9		9/12		15/6	
TNM stage						
I–II	1/9	0.000	8/2	0.020	5/5	N
III–IV	19/4		7/16		18/5	

Abbreviation: N, no significant difference.

Statistical significance is assessed using the Pearson χ^2^ test, Fisher's exact test, or the Mann-Whitney *U* test. Differences are considered statistically significant in a two-tailed test for *p* < 0.05.

### MGMT hypermethylation and expression loss are recapitulated in CagA plasmid transfected or CagA+ HP infected GC cells

**Figure 2:**
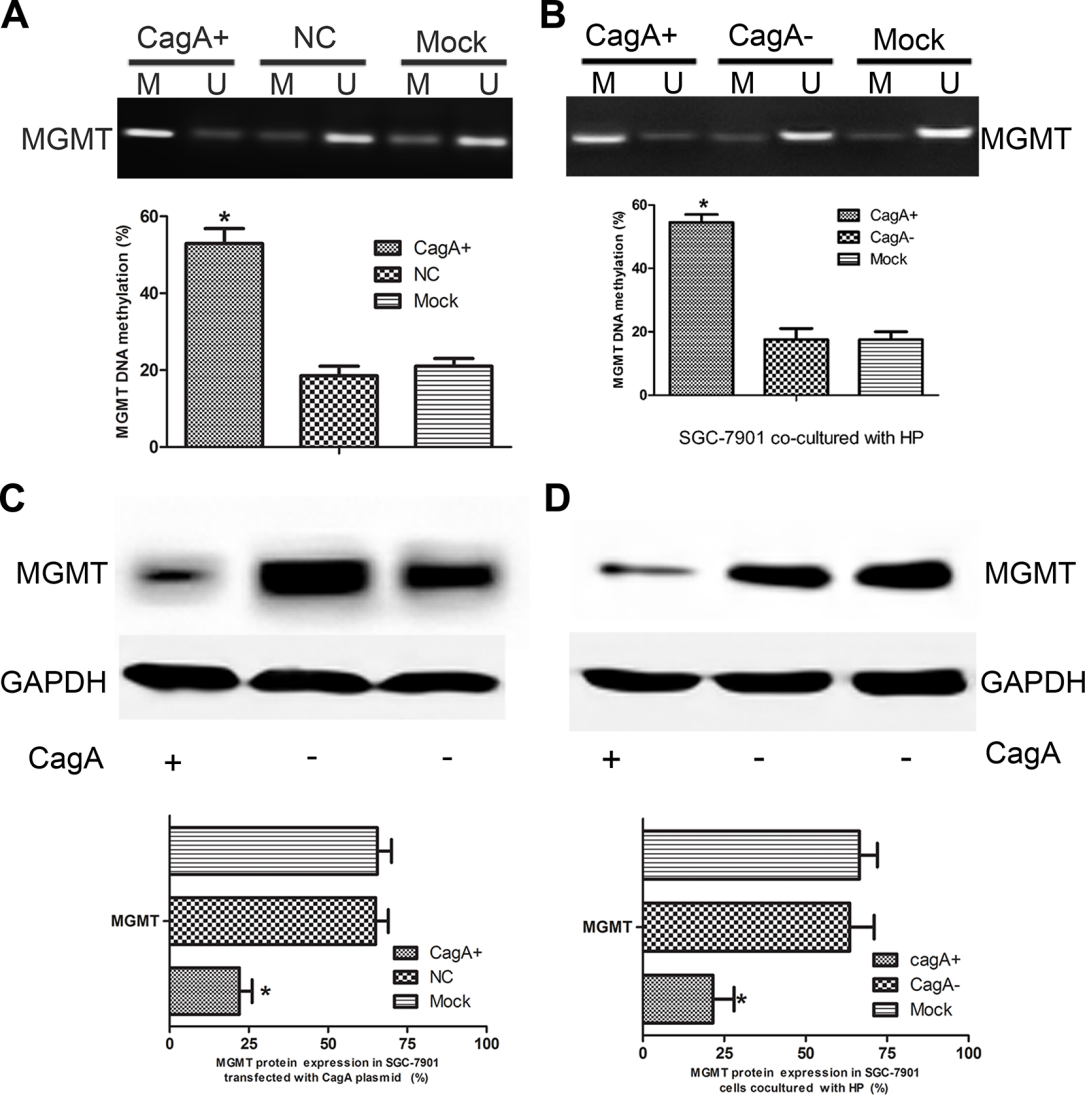
CagA induces MGMT gene hypermethylation and its expression loss (**A** and **B**) *MGMT* methylation status in CagA+ SGC-7901 and HP-infected cells. Increased *MGMT* methylation is observed in CagA transfected and CagA+ HP infected cells compared with control groups. (**C** and **D**) MGMT expression in CagA stablely transfected and HP cocultured (MOI 100:1) cells detected by WB. MGMT downregulation is observed in CagA stablely transfected and CagA+ HP infected cells compared with control groups. The graph represents densitometric analysis of the bands obtained for each signal. Results are expressed as relative expression compared with control cells (*p < 0.05). Each value is the mean ± SD of three experiments.

To further investigate the mechanisms underlying the MGMT loss in HP-associated GC, we successfully constructed stable transfectant: CagA-expressing SGC-7901 cell line (CagA+ SGC-7901), pcDNA3.1 empty vector transfected SGC-7901 (EV-SGC-7901) cell line serves as control. HP infected GC cell line SGC-7901 was established using the co-culture method [13]. The methylation status of *MGMT* was measured by MSP in these cell lines. We found that the MGMT methylation degree indeed increased significantly in the SGC-7901 cell line transfected with CagA plasmid and infected with CagA+ HP (*p* < 0.05) (Figure 2A–2B). Further, MGMT expression also markedly decreased in CagA+ SGC-7901 and cells infected with CagA+ HP (*p* < 0.05) (Figure 2C–2D).

### CagA induced tumor suppressor genes hypermethylation by upregulating DNMT1

**Figure 3:**
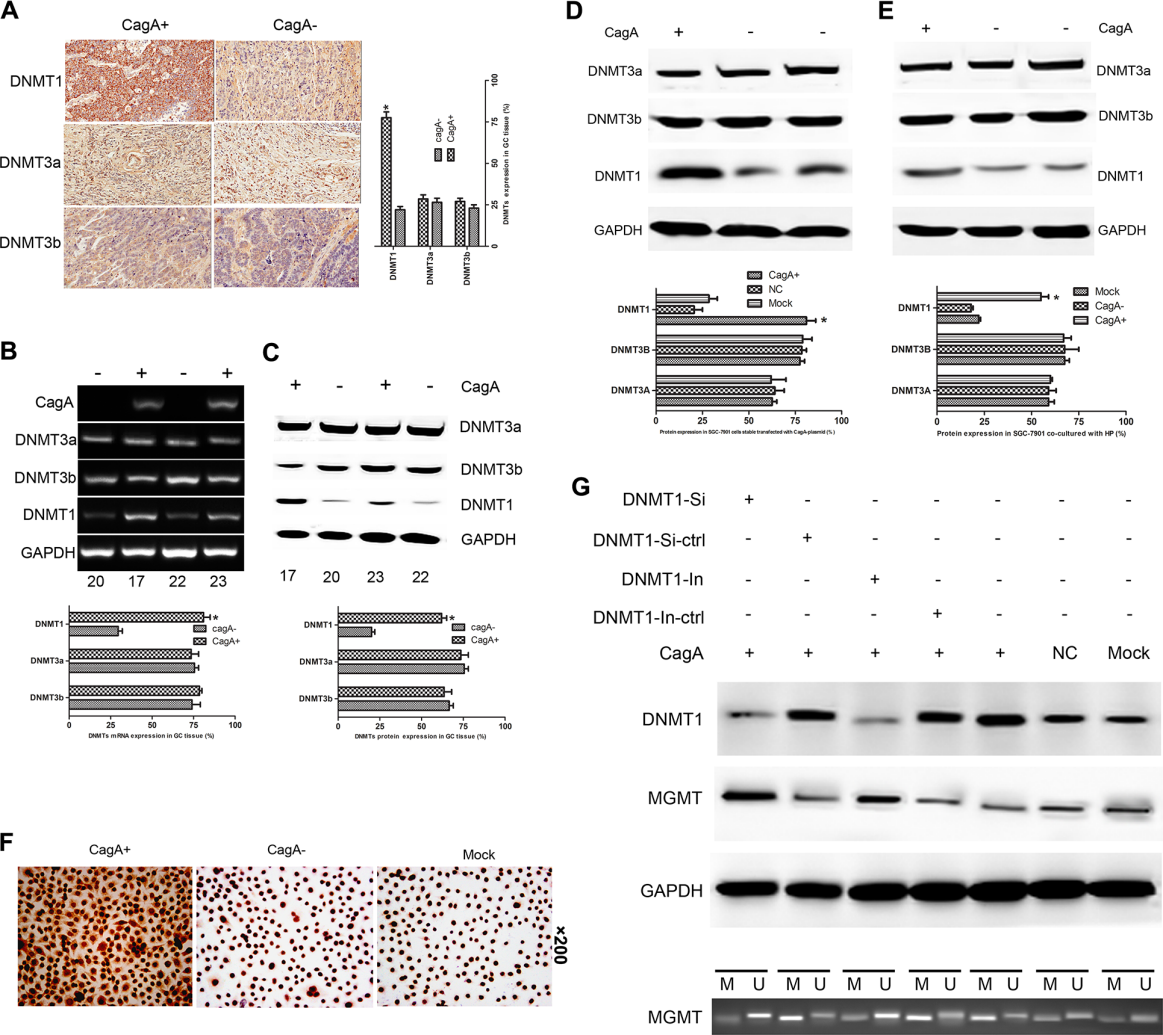
CagA-induced DNMT1 upregulation is responsible for MGMT promoter hypermethylation and its expression loss (**A**) Immunohistochemical staining of DNMTs in HP-associated tissues. A marked elevation of DNMT1 is found in CagA+ tissues compared with CagA− tissues, whereas no difference in DNMT3a or DNMT3b expression is observed between groups. (**B** and **C**) mRNA and protein expression of DNMTs in HP-associated tissues. Consistent with the results shown in (A), there is a significant difference in DNMT1 expression between CagA+ and CagA− tissues, while DNMT3a or DNMT3b expression remains unchanged in different groups. (**D** and **E**) DNMTs expression in CagA+ cells and HP cocultured cells analysed by WB. A remarkable DNMT1 upregulation is found in CagA+ cells and HP cocultured cells compared with control groups while no difference of DNMT3a and DNMT3b exists between groups. (**F**) Immunocytochemical analysis of DNMT1 expression in CagA+ SGC-7901. A much stronger positive reaction is confirmed in CagA+ cells compared with control groups. (**G**) MGMT hypermethylation is reversed by DNMT1 inhibition. (UP) DNMT1 and MGMT expression is reversed by siRNA or inhibitor (5-aza-cdr) of DNMT1. (DOWN) MGMT hypermethylation is reversed by DNMT1 siRNA or its inhibitor. The graph represents densitometric analysis of the bands obtained for each signal. Results are expressed as relative expression compared with control cells (*p < 0.05). Each value is the mean ± SD of three experiments.

Since activation of any DNMT protein is considered a potentially significant mean of causing CpG island methylation in human carcinomas [18], we evaluated the expression of representative DNMT proteins (DNMT1, DNMT3a and DNMT3b) in HP associated GC tissues, CagA transfected and HP cocultured cells respectively. A marked increase of DNMT1 expression was observed in CagA^+^ HP infected GC tissues (Figure 3A–3C and Table 1), CagA transfected (Figure 3D and 3F) and CagA^+^ HP cocultured (Figure 3E) cells (*p* < 0.05). In contrast, no difference in the expression of DNMT3a or DNMT3b was observed in different groups of HP associated tissues and cell lines. To further confirm whether the CagA induced MGMT promoter hypermethylation was dependent on DNMT1 upregulation, we used siRNA or protein inhibitor to inhibit DNMT1, and found that MGMT hypermethylation and MGMT expression reduction could be reversed by DNMT1 siRNA or its specific inhibitor (5-aza–cdr) (*p* < 0.05) (Figure 3G).

### CagA enhanced DNMT1 expression via PDK1/AKT-NFκB pathway

**Figure 4:**
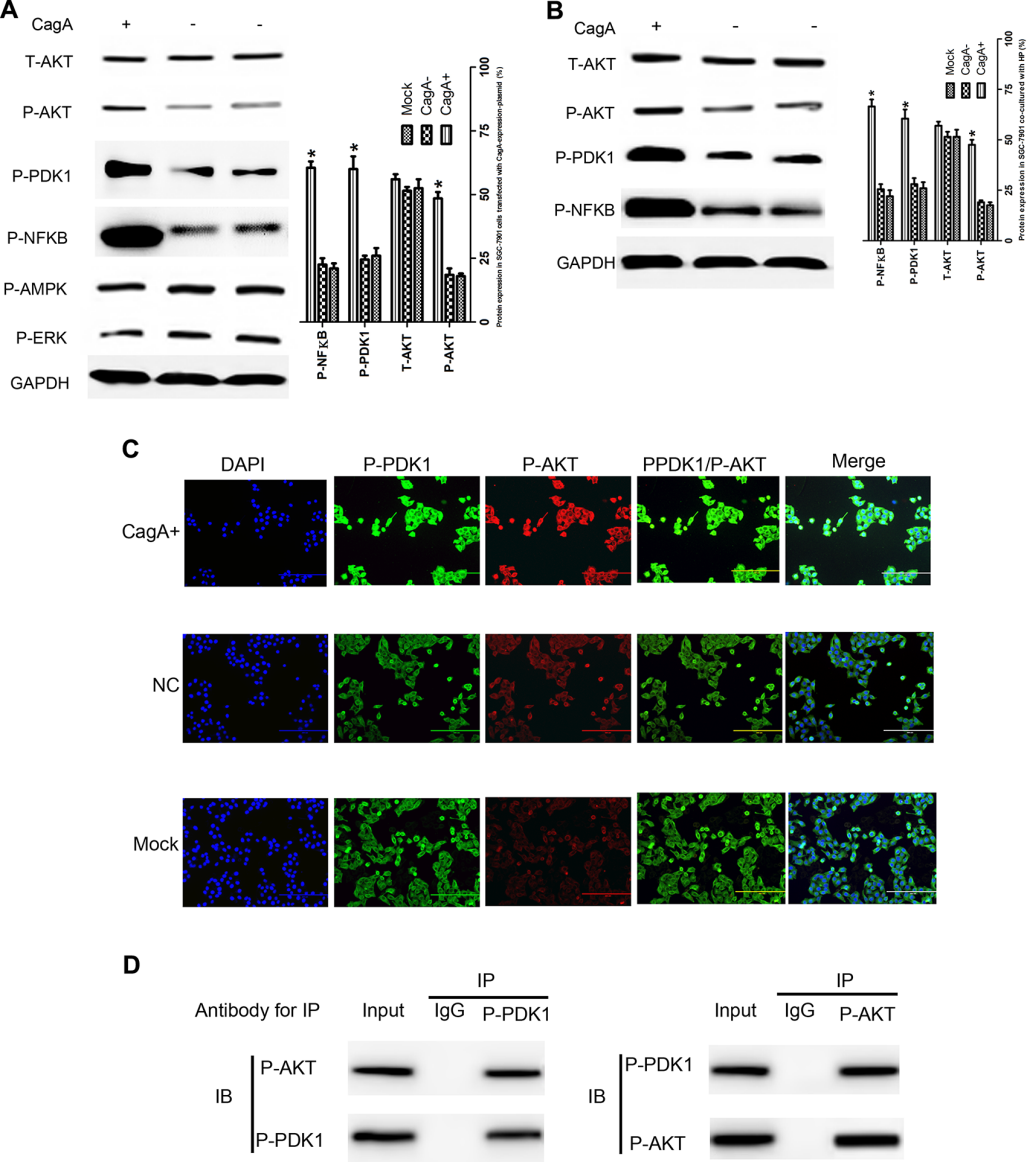
CagA activates AKT-NFκB pathway by enhancing the interaction of P-PDK1 and P-AKT (**A** and **B**) GC related signal proteins expression in differently treated cells detected by WB. P-PDK1/P-AKT/P-NFκB increases in CagA transfected or HP (MOI 100:1) cocultured cells compared with control cells. (**C**) Co-localization of P-PDK1 and P-AKT. The intracellular co-localization of P-PDK1 and P-AKT is analyzed using immunofluorescence microscopy. The overlay is indicated by yellow color. Data shows that the CagA group has a strong yellow signal which representes tightly interaction of P-PDK1 and P-AKT. (**D**) The IP test confirms the interaction of P-PDK1 and P-AKT. Data shows that P-PDK1 and P-AKT protein could be precipitated by mutual antibodies. These data suggest that CagA enhances the interaction of P-PDK1 and P-AKT. The graph represents densitometric analysis of the bands obtained for each signal. Results are expressed as relative expression compared with control cells (*p < 0.05). Each value is the mean ± SD of three experiments.

Till now, no evidence revealed that CagA could act as a transcription factor to regulate gene expression directly, so we presumed that CagA increased DNMT1 expression by disdurbing some key signal pathway in GC. Since AKT has been well known to play a significant role in regulating genes expression and the aforementioned data revealed that P-PDK1 is a known activator and binding partner of AKT [19], also, it's well established that NFκB is a downstream element of the AKT pathway [20], so we investigated whether PDK1/AKT-NFKB pathway is involved in DNMT1 upregulation and subsequent MGMT hypermethylation. To this end, the total and phosphorylation protein of PDK1/AKT-NFκB pathway in the nucleus and cytoplasm of cells differently treated were detected. Indeed, we found that P-PDK1, P-AKT and P-NFκB expression significantly increased both in CagA transfected and CagA+ HP infected cells (Figure 4A–4B), while there was no significant difference of other signal proteins existed between groups.

**Figure 5:**
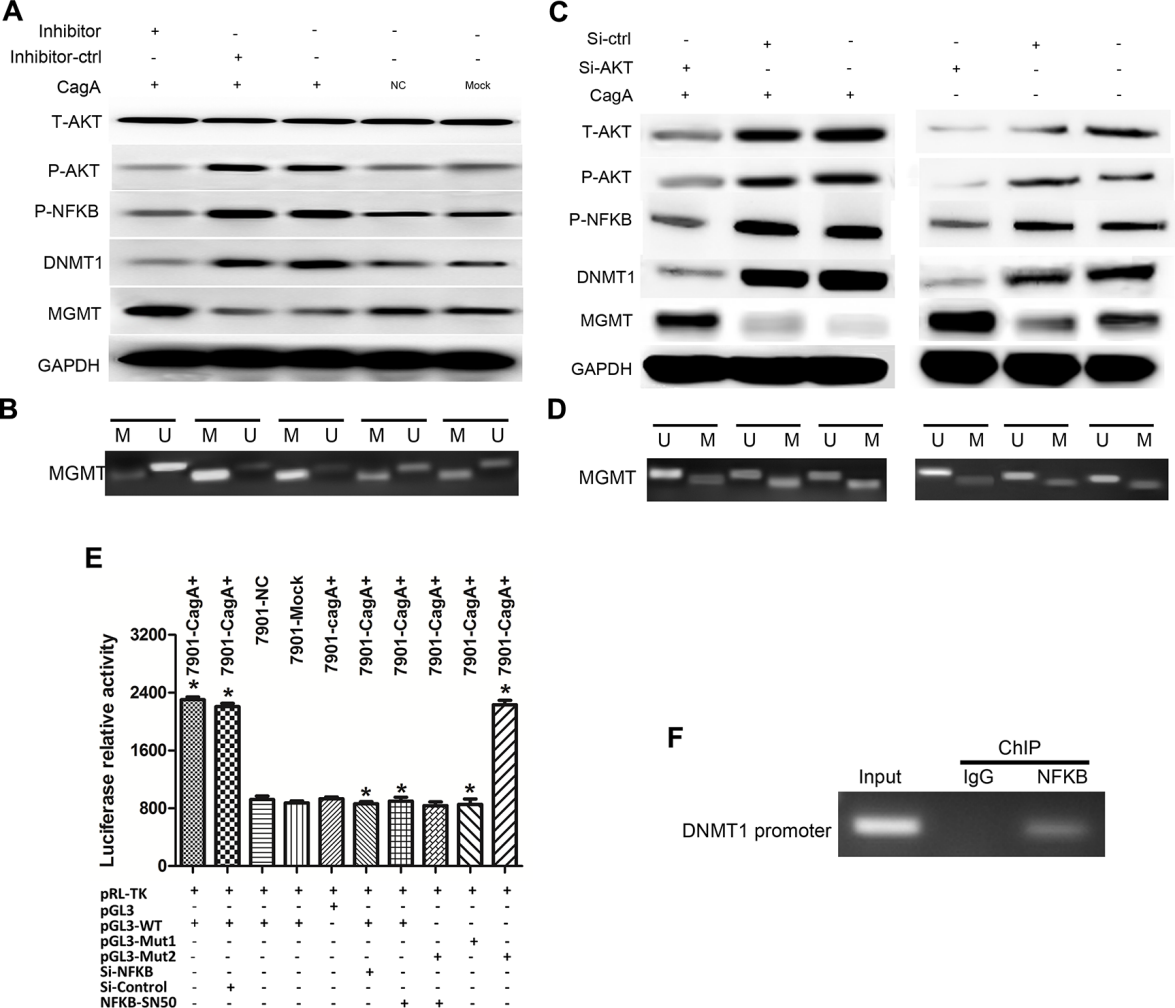
CagA-mediated DNMT1 upregulation is dependent on constitutive AKT phosphorylation and subsequent activated NFκB which combines to DNMT1 promoter (**A** and **C**) WB analysis of related signal proteins in different groups after treated with AKT inhibitor (MK-2206 2HC) or AKT siRNAs. MGMT reduction in CagA+ SGC-7901 is reversed by AKT inhibitor or AKT siRNAs, On the other hand, T-AKT, P-PDK1/P-AKT/P-NFκB and DNMT1 expression significantly decrease in CagA+ cells compared with control cells. (**B** and **D**) MSP analysis of *MGMT* methylation in CagA+ SGC-7901 or EV-SGC-7901 after AKT inhibiton. *MGMT* hypermethylation is also markedly reversed in CagA+ SGC-7901 by AKT inhibitor or AKT siRNAs compared with control cells. (**E**) Reporter plasmids of DNMT1 (pGL3-DNMT1) are generated by ligating DNMT1 promoter region into the pGL3-basic vector. CagA transfection significantly increases luciferase activity, which is significantly reduced by NFκB inhibition. (**F**) DNMT1 promoter region and NFκB interaction is validated by the ChIP. Data shows that DNMT1 DNA is detectable in the ChIP sample of CagA+ SGC-7901 using an antibody against NFκB1 p50, suggesting that NFκB1 p50 binds to DNMT1 promoter. These data suggest that CagA increased DNMT1 expression may depend on NFκB activation. Results are expressed as relative expression compared with control cells (*p < 0.05). Each value is the mean ± SD of three experiments.

To elucidate how CagA promoted AKT phosphorylation, we used immunofluorescence or IP analysis and found that P-PDK1/P-AKT interaction in CagA transfected cells increased sharply (Figure 4C–4D). To confirm the correlation between AKT phosphorylation, DNMT1 upregulation and subsequent MGMT hypermethylation, we inhibited AKT activity by its specific inhibitor or siRNAs, and found that DNMT1 expression decreased while MGMT expression subsequently increased in parallel with P-AKT reduction (Figure 5A and 5C). Furthermore, *MGMT* methylation significantly decreased in parallel with P-AKT reduction (*p* < 0.05) (Figure 5B and 5D). Thus, P-AKT and DNMT1 overexpression closely correlated with *MGMT* hypermethylation and its expression reduction. Therefore, these results are consistent with the proposal that DNMT1, upregulated by CagA in a P-AKT dependent manner, promoted *MGMT* hypermethylation, leading to MGMT reduction in GC. In order to determine whether NFκB regulated DNMT1 directly, the firefly luciferase reporter constructs containing DNMT1 promoter with NFκB potential target sites was generated (DNMT1-WT/MT1/MT2) and transfected into GC cells, and we found that the luciferase activity increased significantly in cells co-transfected with CagA and DNMT1-WT or DNMT1-MT2 compared with control cells. Consistent with this finding, the luciferase activity was significantly decreased by NFκB inhibitor, SN50, or siRNA. Also, no difference in the luciferase activity between DNMT1-MT2 and DNMT1-WT groups was observed, which indicated that MT2 location may not contain NFκB binding sites (Figure 5E). To confirm these results, we employed ChIP and found that NFκB could bind to the DNMT1 promoter region (Figure 5F). In total, these data strongly suggested that NFκB could up-regulate DNMT1 expression by binding to its promoter directly.

### CagA induced DNMT1 up-regulation, subsequent *MGMT* hypermethylation and MGMT loss via PDK1/AKT-NFκB pathway reoccurred *in vivo*

**Figure 6:**
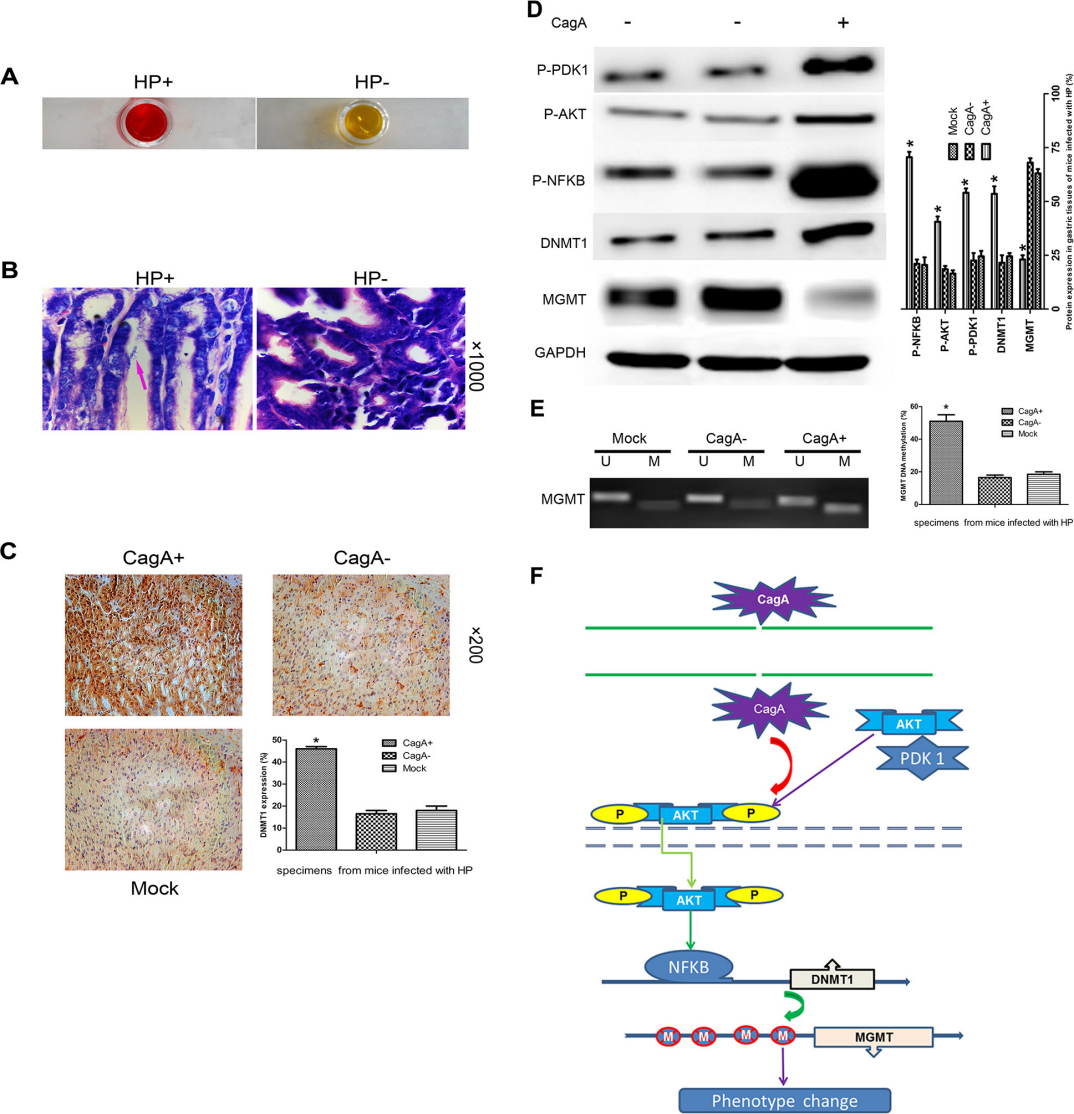
CagA-mediated MGMT hypermethylation and subsequent protein decrease by upregulating DNMT1 via PDK1/AKT-NFκB pathway is recapitulated *in vivo* (**A** and **B**) RUT and Giemsa staining validates the successful HP colonization in C57BL/6 mice. (**C**) IHC staining of gastric tissues from mice infected with different HP strains. A marked DNMT1 elevation occurs in CagA+ HP infected tissues compared with control group. (**D**) WB analysis of related protein expression in mouse gastric tissues infected with different HP strains. A notable MGMT loss paralleled with a significant increase in P-PDK1/P-AKT/P-NFκB/DNMT1 expression is observed in CagA+ HP infected tissues compared with control groups. (**E**) MSP analysis *MGMT* methylation in gastric tissues from mice infected with different HP strains. A significant increase in *MGMT methylation* is observed in CagA+ HP infected tissues compared with control groups, which is consistent with what has been confirmed *in vitro*. (**F**) Signal pathway schematic diagram. CagA is delivered into the cytoplasm by a type IV secretion system of the adhered HP. CagA then causes an increase in AKT phosphorylation by activating PDK1, subsequently, activated P-AKT translocates into the nucleus where it upregulates DNMT1 by activating NFκB. The upregulated DNMT1 further promotes *MGMT* hypermethylation, which leads to MGMT loss. The graph represents densitometric analysis of the bands obtained for each signal. Results are expressed as relative expression compared with control cells (*p < 0.05). Each value is the mean ± SD of three experiments.

To determine if the above results could be recapitulated *in vivo*, we performed the following experiment: Six to eight weeks old C57BL/6 mice were orally inoculated once with 1 × 10^**8**^ CFU HP strain ATCC49503 (CagA+) or ATCC51932 (CagA−). Control mice were not given bacteria. Mice were sacrificed four months after inoculation and gastric tissues were collected. The mice that had been successfully infected with HP were confirmed by RUT or Giemsa staining (Figure 6A–6B). As observed in clinical tissues, HP co-cultured and CagA transfected cells, a significant up- regulation of P-PDK1, P-AKT, P-NFκB, or DNMT1 and MGMT down-regulation were observed *in vivo* (*p* < 0.05) (Figure 6D). In addition, IHC staining showed that DNMT1 expression elevated, which was in accordance with WB results (Figure 6C). We then evaluated the *MGMT* methylation using MSP, and as shown in Figure 6E, a marked increase in *MGMT* methylation in tissues infected with CagA+ HP strain was observed compared with CagA− HP strain (*p* < 0.05).

In total thus, our results are consistent with the following model of CagA-mediated aberrant *MGMT* methylation in GC tissues (Figure 6F): The adherence of HP to gastric epithelia results in the delivery of CagA into the cytoplasm, where it activates AKT via P-PDK1. Subsequently, P-AKT translocates into the nucleus where it activates NFκB which upregulates DNMT1. Finally, the increased DNMT1 results in *MGMT* hypermethylation, causing its reduced expression, which contributes to the initiation and development of GC.

## DISCUSSION

A causal relationship between HP and GC is first postulated by Marshall and Warren in 1983 [1]. More and more data shows that oncoprotein CagA is the most important molecule among HP virulent factors, upon HP attachment to the epithelial cell, the CagA is injected directly into the cell via the TFSS [5]. CagA then localizes to the plasma membrane inner surface, where it undergoes tyrosine phosphorylation by Src family kinases (SFK) such as c-Src [21]. Accordingly, upon phosphorylation, CagA disturbs signal transduction, and thereby provokes cellular dysfunction that eventually leads to cell transformation [22].

Carcinomas showing a high degree of tumor suppressor gene CpG island methylation are referred to as a CpG island methylator phenotype (CIMP), which is the most characteristic among genetic and epigenetic abnormalities in HP-associated GC [23]. Niwa *et al.* showed specific types of chronic inflammation (HP infection) are necessary for methylation patterns changes [24]. Tumor suppressor genes methylation that occurs in a number of mucosa cells establishes an ‘epigenetic field for cancerization’ or an ‘epigenetic field defect’, which distinguishes a site as a high risk for subsequent malignant transformation [25]. Previous study has noted that methylated MGMT is significantly associated with CagA+ HP infection (*p* < 0.035) [14]. Consistent with this, in this study, we found that the MGMT gene represents an example of this typical feature. *MGMT* promoter methylation and MGMT expression repression were observed concurrently in CagA-associated GC tissues, GC cell lines stablely transfected with CagA or infected with CagA^+^HP.

Almost all of the East Asian HP strains are cag-PAI-positive [3]. There is compelling evidence to support the importance of HP in inducing tumor suppressor genes methylation in GC development but less is known about the mechanisms. The key enzymes mediated hypermethylation mainly include DNMTs [17]. Our data shows that DNMT1 upregulation positively correlated with CagA in GC specimens. And there is growing evidence that many kinds of viral proteins could regulate DNMT1 expression via different signal pathways [26–28]. EBV-LMP2A activates DNMT1 transcription and causes a reduction of PTEN expression through CpG island methylation of the *PTEN* promoter in GC [29]. The hypermethylation of the PTEN promoter was also observed in our study (Figure 1C). It has been shown that *p16* inactivation induced by CpG islands methylation could be gradually restored within about 2 weeks after treatment with a DNMT1 inhibitor or after HP eradication [30]. To clarify the mechanism of CpG island methylation, we utilized HP-infected and CagA stably transfected GC cell lines. In this way, CagA proved to be responsible for the upregulation of DNMT1 in stomach epithelial cells. The intracellular function of CagA and its modulation of signaling pathways of hypermethylation have not yet been fully clarified in GC.

Till now, no data shows that CagA could act as a transcpription factor to regulate gene expression directly, so we presumed that CagA increased DNMT1 via some key signaling pathways, such as AKT-NFκB [31]. Our data clearly showed that AKT phosphorylation significantly increased with CagA expression or HP stimulation, which is consistent with a recent study [31]. Further, we found that CagA could activate PDK1, an essential component in the activation of AKT, and strengthen its interaction with AKT as a key downstream molecule of the AKT pathway, NFκB plays an important role in carcinogenesis [20]. We thus evaluated the function of NFκB in DNMT1 up-regulation induced by CagA, and found that NFκB could indeed be activated following AKT phosphorylation. Moreover, we showed that NFκB directly promoted the DNMT1 expression after activation by CagA. Our findings suggest that CagA induced MGMT hypermethylation by upregulating DNMT1 dependent on PDK1/AKT-NFκB pathway, which was confirmed *in vivo*. Further studies are necessary to determine to what extent the CagA-AKT-NFκB-DNMT1 pathway contributes to the global CpG island methylation of tumor suppressor genes in HP-associated GC *in vivo* and what conditions make it specific among other AKT targets.

In conclusion, this study has shown that CagA induced the phosphorylation of AKT by enhancing its interaction with PDK1, which then led to DNMT1 overexpression via NFκB activation. We speculate that this increased methylation activity leads to an accumulation of tumor suppressor hypermethylation, such as MGMT, eventually surpassing a threshold whereby the normal integrity of stomach epithelial cells is compromised and thus playing a significant role in the overall GC development. Thus, CagA-AKT/NFκB-DNMT1 pathway maybe a potential therapy target of HP-associated GC.

## MATERIALS AND METHODS

### Tissues and cell lines

Tumor tissues and its paired adjacent non-tumor tissues of GC were collected from the patients underwent radical gastrectomy for GC at Ruijin Hospital between 2012 and 2014. Tissue slices were taken from 10% formalin-fixed, paraffin-embedded tissue blocks. All GC cases were histologically diagnosed according to the Japanese Classification of Gastric Carcinoma and Lauren's classification [32, 33]. Human GC cell line SGC-7901 was purchased from Shanghai Institutes for Biological Sciences, Chinese Academy of Sciences. The cells were cultured routinely in RPMI-1640 supplemented with 10% heat-inactivated fetal bovine serum (FBS), 100 U/ml penicillin and 100 μg/ml streptomycin in a humidified cell incubator with an atmosphere of 5% CO_2_ at 37°C. Exponentially growing cells were used for experiments.

### H. pylori strains and cell co-culture

H pylori bacterial strains 60190 ((ATCC 49503, CagA+) and Tx30a (ATCC 51932, CagA−) were got from American Type Culture Collection. Bacteria were routinely cultured on 5% horse blood agar plates (Oxoid Ltd, Basingstoke, UK) in humidified incubators, which provided an atmosphere of 5% CO_2_ at 37°C. GC cells were trypsinized, resuspended in normal growth medium and seeded into 6 well plates. Three duplicate wells were prepared for each experimental condition. When the cells reached 70% confluence they were serum-starved for 24 h prior to the addition of HP at a multiplicity of infection of 100. Cells were co-cultured with the bacteria for 24 h before either RNA or DNA extraction or protein measurements were performed.

### Detection of HP infection

Rapid Urease Test (RUT) and Giemsa staining were employed for detecting HP infection. The presence of HP in tissues was evaluated using a RUT-targeting HP-encoded urease kit(Shanghai Hui Tai Medical Science and Technology Co., Ltd). If the gel became pink, red or dark red within 5 min, this signified a positive result, and it's a negative result if the gel remained yellow. Giemsa staining was carried out according to the manufacturer's instructions (Gibco, CA, USA). HP infection was diagnosed as positive if one of the methods produced positive result.

### Establishment of stable transfectants

The CagA plasmid was prepared from the amplified product of HP *CagA* gene. After digestion with SpeI and HindIII, the restricted fragment was inserted into pcDNA3.1 vector which was purchased from NOVOBIO. Cells were plated at a density of 2 × 10^5^ cells/cm^**2**^, cultured for 24 h and then stably transfected with CagA pcDNA3.1 or empty pcDNA3.1 vectors. After 24 h, and every 48 h thereafter for 4 weeks, culture medium was replaced with fresh medium containing 800 μg/ml of G418. Pools of 16 clones were isolated as stable transfectants with a CagA vector (CV) and an empty vector (EV) in SGC-7901 cells. Individual clones were isolated for further study.

### Immunohistochemistry (IHC), western blotting (WB) and immunocytochemistry (ICC)

For IHC, sections were de-waxed using three changes of xylene for 4 min each and microwave heated to 98°C for 15 min (EMS900, Electron Microscopy Sciences, Hatfield, PA, USA) in citric acid buffer (pH 6.0), then incubated with primary antibodies (Abcam, Cambridge, UK) for 1 h. Secondary (Dako) antibodies were applied for another hour following the removal of the primary antibodies. Staining was developed with an avidin biotinylated horseradish peroxidase complex and DAB (Dako). Expression of MGMT, DNMT1, DNMT3a and DNMT3b was detected in both the cytoplasm and nucleus and they were considered overexpression if more than 15% cells were stained positive. For WB, following trypsinization, cell pellets were washed with PBS and cell extracts were prepared using sonication in 50 mM Tris-HCl buffer (pH 8.0) containing 1% glycerol, 1 mM EDTA, 0.5 mM PMSF and 2 mM benzamidine followed by centrifugation. Equal protein amounts (100 μg) were electrophoresed on 12% reducing SDS-polyacrylamide gels. Proteins were electrotransferred to Immobilon-P membranes. Membranes were blocked with 5% non-fat dry milk in Tris-buffered saline (TBS; pH 8.0) containing 0.1% Tween-20 for 2 h, and subsequently incubated with primary antibodies (Abcam, Cambridge, UK) at 1 μg/ml. Antigen–antibody complexes were visualized using enhanced chemiluminescence. Band intensities were quantitated using a Tanon 2500 imaging system (TANON). For ICC, cells were grown in 6-well plates containing sterile cover slips. When cells reached ≤ 80% confluence, the medium was removed from and cells were fixed in 2% paraformaldehyde for 20 min before they were permeabilized with 0.1% Triton-X100 for 5 min and blocked for 1 h at room temperature. The primary antibody was applied (dilution 1:200) on cells and incubated at 4°C overnight. The next day, secondary antibodies were applied for another hour after the primary antibodies removal. Staining was developed with DAB. Cells were washed with 1 × PBST between steps.

### Immunofluorescence analysis

Cells on coverslips were washed twice with PBS and fixed in 2% paraformaldehyde at room temperature for 15 min. Cell Permeabilization was performed with 0.1% Triton in PBS at 4°C for 10 min. The cells were blocking with 2% BSA at room temperature for 1 h, and rinsed three times with PBS before incubated with primary antibody at 4°C overnight. Cells were incubated with a secondary antibody for 2 h before immunofluorescence detection. Nucleus was stained with DAPI for 3 min. Images were acquired using an immunofluorescence microscopy (Olympus) equipped with 20 × objective.

### Immunoprecipitation

Immunoprecipitation was employed using a co-IP kit (Pierce) as the manufacturer's instructions. Cells were lysed at 4°C in 50 mmol/L Tris-HCl pH = 7.5, 150 mmol/L NaCl, 1% Brij-96, 0.1% sodium dodecyl sulfate, 10 mmol/L NaF, 5 mmol/L VO4, and protease inhibitors. 300 μg proteins were incubated with 3 μg primary antibodies at 4°C overnight. Immune complexes were precipitated with protein A/G sepharose beads and washed extensively before WB analysis.

### Luciferase reporter assay

We analyzed DNMT1 promoter region for the potential NFκB binding site using the web sites (http://alggen.lsi.upc.es/cgi-bin/promo_v3/promo/promoinit.cgi?dirDB=TF_8.3 and http://jaspar.genereg.net/). The promoter of human DNMT1 gene was cloned into the pGL3 reporter plasmid (Ambion), obtaining the WT firefly luciferase reporter gene. Overlap extension PCR was applied to introduce mutations into the seed sequences of NFκB binding sites and generate the DNMT1 MT1 and MT2 reporter genes, which mutated at positions −856 to −867 and −1103 to −1114, respectively. Cells seeded onto 24-well plates were co-transfected with firefly reporter constructs containing the DNMT1 promoter, Renilla expressing plasmid, pRL-TK, and CagA plasmid or control plasmid using Lipofectamine 2000. Firefly luciferase activity and Renilla luciferase activity were measured 24 h after the initiation of transfection by the Dual Luciferase Asssay Systems (Promega). Firefly luciferase activity was normalized to Renilla luciferase activity.

### Chromatin immunoprecipitation (ChIP) assay

To evaluate the effect of CagA on the interaction between NFκB and DNMT1 promoter region, ChIP was performed as the manufacturer's instructions (millipore). Cells were fixed with formaldehyde for protein/DNA crosslinking and lysed. The DNA was sheared by sonication (15 pulses, 35 sec on 35 sec off) and added to a well coated with the anti-NFκB (Abcam). Washes were performed to remove unbound material, while NFκB-bound DNA was released by protein digestion with proteinase K. The DNA was purified through a column and PCR was performed using primers designed to target the DNMT1 promoter region spanning the site of interaction with NFκB, while genomic DNA and IgG were used as control.

### DNA extraction, methylation-specific PCR (MSP), RNA isolation and RT-PCR

Genomic DNA was isolated using a QIA-amp DNA Mini Kit (QIAGEN) according to the manufacturer's instructions. *MGMT* methylation status was determined using a bisulfite modification and MSP assay with the Epi-Tect Bisulfite Kit and Epi-Tect MSP Kit (QIAGEN). The products were run on a 2% agarose gel and analyzed with DNA-GREEN staining. RNA was extracted with TRIzol reagent (Invitrogen) and 1 μg RNA was reverse transcribed using an oligo (dT) primer and RTAMV reverse transcriptase (Invitrogen, Carlsbad, CA). The resulting cDNA was amplified under the following conditions: 2 min 95°C for the first cycle; 95°C for 1 min, 60°C for 55 s, and 72°C for 2 min for the next 35 cycles; and a final elongation step at 72°C for 10 min. RT-PCR products were separated using electrophoresis on a 1.5% agarose gel stained with DNA-GREEN (PCR grade), and visualized using UV light.

### Plasmid and siRNA transfection and inhibitor application

siRNA (750 pmol) (Santa Cruz, CA, USA) was transfected into cells (2 × 10^6^) using the Lip2000. After transfection, cells were cultured in RPMI-1640 supplemented with 10% heat-inactivated FBS. Plasmid DNA and lipofectamine were diluted separately in serum-free medium and incubated at room temperature for 5 min. After incubation, the diluted DNA and Lipofectamine were mixed and incubated at room temperature for 20 min. Aliquots of the transfection mixture were added to each well of the cell culture plate. Inhibitors were used as follows: MK-2206 2HC (Selleck) 15 nM for AKT, 5-aza-cdr (Sigma) 0.2 μM for DNMT1, SN50 (Merck) 36 μM for NFκB.

### HP infection *in vivo*

Six to eight-week-old C57BL/6 mice (Institute of Zoology, Chinese Academy of Sciences, Shanghai, China) were inoculated orally once with 1 × 10^**8**^ CFU of broth-cultured HP strains 60190 (CagA +) or Tx30a (CagA−), in 0.15 ml broth. Control mice were given PBS. Mice were sacrificed four months after inoculation and gastric tissues were collected for HE, RT-PCR, WB and MSP analysis.

### Ethics statement

All animal experiments described in this article were conducted according to China guidelines for animal experimentation and approved by the Institutional Animal Care Committee of Ruijin Hospital affliated Shanghai Jiao-Tong University School of Medicine. All patients provided written informed consent before enrollment and the Ethic Committee of Ruijin Hospital Shanghai Jiao-Tong University School of Medicineapproved the study protocol.

### Statistical methods

Statistical analyses were performed using a Student's *t*-test or one-way ANOVA, the chi-squared test, Fisher's exact test or the Mann-Whitney *U* test. Differences were considered statistically significant in a two-tailed test for *p*-values < 0.05. The statistical analysis software used was SPSS Version 19.0.

## SUPPLEMENTARY MATERIALS TABLE


