# Supplementary material for: Helicobacter pylori CagA induces tumor suppressor gene hypermethylation by upregulating DNMT1 via AKT-NFκB pathway in gastric cancer development
Source: Oncotarget. 2016 Feb 2;7(9):9788–800. doi: 10.18632/oncotarget.7125 (PMC4891084; doi:10.18632/oncotarget.7125)
Supplement: Supplementary file 1 [file oncotarget-07-09788-s001.pdf]

# Helicobacter pylori CagA induces tumor suppressor gene hypermethylation by upregulating DNMT1 via AKT-NFκB pathway in gastric cancer development

## Supplementary Materials

### Supplementary Table S1: Primer sequences used in the present study

| GeneSequence5,–3, | |
|---|---|
| GAPDH-F | CTCTGCTCCTCCTGTTCGAC |
| GAPDH-R | TTAAAAGCAGCCCTGGTGAC |
| CagA-F | GATAACAGGCAAGCTTTTGAGG |
| CagA-R | CTGCAAAAGATTGTTTGGCAGA |
| Dnmt1-F | TACCTGGACGACCCTGACCTC |
| Dnmt1-R | CGTTGGCATCAAAGATGGACA |
| Dnmt3a-F | TATTGATGAGCGCACAAGAGAGC |
| Dnmt3a-R | GGGTGTTCCAGGGTAACATTGAG |
| Dnmt3b-F | GGCAAGTTCTCCGAGGTCTCTG |
| Dnmt3b-R | TGGTACATGGCTTTTCGATAGGA |
| MGMT-F | ATGGACAAGGATTGTGAAATG |
| MGMT-R | GAAAACGGGATGGTGAAGAGC |
| MGMT-u-F | GTGTAGTTGTTTTGAGTAGGATTGG |
| MGMT-u-R | ACCTTAATTTACCAAATAACCCATA |
| MGMT-m-F | CGTAGTCGTTTCGAGTAGGATC |
| MGMT-m-R | ACCTTAATTTACCAAATAACCCGTA |
| P16-u-F | TTATTCGCGGGTGGGGTGTT |
| P16-u-R | CAACCCCAAACCACAACCAA |
| P16-m-F | TTATTAGAGGGTGGGGCGC |
| P16-m-R | GACCCCGAACCGCGACCGA |
| hmlH1-u-F | GTGTTTTGATGTTTGTAGGT |
| hmlH1-u-R | TCCACACTCTTCCAAAAACA |
| hmlH1-m-F | TTTCGATTCGTAGGTTCGCC |
| hmlH1-m-R | GCACTCTTCCGAAAACGAAG |
| PTEN-u-F | GAGAGATGGTGGTGGTTGT |
| PTEN-u-R | AACTCCCCAAAAACACTACC |
| PTEN-m-F | GGCGGCGGTCGCGGTTC |
| PTEN-m-R | GACTCCCCGAAAACGCTAC |
| DNMT1-WT-F | TGAGGGGCTTTCCTATCCTGT |
| DNMT1-WT-R | TTCAGGTGCTGCCCTGTTTC |
| DNMT1-MT1-F | ATGACCCCGAAAGGAATCCTGTTGTCGAACTGCTGGTTACACACACACACA |
| DNMT1-MT1-R | GTTCGACAACAGGATTCCTTTCGGGGTCATTCCAGCAACAAAATTTTTTTG |
| DNMT1-MT2-F | GGGGAGTCCCCCAAGGGAAACAGGGCAGCACCTGAATTCTGGAGGTCC |
| DNMT1-MT2-R | TGCCCTGTTTCCCTTGGGGGACTCCCCGTGAGGTCACTCTTTCCTGCC |
